# Loop-Mediated Isothermal Amplification (LAMP) Assay for Detecting *Burkholderia cepacia* Complex in Non-Sterile Pharmaceutical Products

**DOI:** 10.3390/pathogens10091071

**Published:** 2021-08-24

**Authors:** Soumana Daddy Gaoh, Ohgew Kweon, Yong-Jin Lee, John J. LiPuma, David Hussong, Bernard Marasa, Youngbeom Ahn

**Affiliations:** 1 Division of Microbiology, National Center for Toxicological Research, U.S. Food and Drug Administration, Jefferson, AR 72079, USA; Soumana.daddy-gaoh@fda.hhs.gov (S.D.G.); oh-gew.kweon@fda.hhs.gov (O.K.); 2 Department of Natural Sciences, Albany State University, Albany, GA 31707, USA; yong.lee@asurams.edu; 3 Department of Pediatrics, University of Michigan, Ann Arbor, MI 48109, USA; jlipuma@med.umich.edu; 4 Eagle Analytical Services, Houston, TX 77099, USA; David.Hussong@outlook.com; 5 Office of Pharmaceutical Quality, Center for Drug Evaluation and Research, U.S. Food and Drug Administration, Silver Spring, MD 20993, USA; Bernard.Marasa@fda.hhs.gov

**Keywords:** *Burkholderia cepacia* complex, loop-mediated isothermal amplification (LAMP), nuclease-free water, antiseptics

## Abstract

Simple and rapid detection of *Burkholderia cepacia* complex (BCC) bacteria, a common cause of pharmaceutical product recalls, is essential for consumer safety. In this study, we developed and evaluated a *ribB*-based colorimetric loop-mediated isothermal amplification (LAMP) assay for the detection of BCC in (i) nuclease-free water after 361 days, (ii) 10 μg/mL chlorhexidine gluconate (CHX) solutions, and (iii) 50 μg/mL benzalkonium chloride (BZK) solutions after 184 days. The RibB 5 primer specifically detected 20 strains of BCC but not 36 non-BCC strains. The limit of detection of the LAMP assay was 1 pg/μL for *Burkholderia cenocepacia* strain J2315. Comparison of LAMP with a qPCR assay using 1440 test sets showed higher sensitivity: 60.6% in nuclease-free water and 42.4% in CHX solution with LAMP vs. 51.3% and 31.1%, respectively, with qPCR. These results demonstrate the potential of the *ribB*-based LAMP assay for the rapid and sensitive detection of BCC in pharmaceutical manufacturing.

## 1. Introduction

The genus *Burkholderia* comprises >100 species of Gram-negative, non-spore-forming β-*proteobacteria*. Within this genus, the *Burkholderia cepacia* complex (BCC) includes 24 closely related species of opportunistic pathogens that are commonly found in natural environments [1,2,3,4]. These species may contaminate various drug products and are a public health concern. A report published in 2007 [5] surveying the U.S. Food and Drug Administration (FDA) database from 1998 to 2006, documented that ‘*B. cepacia*’ accounted for 22% of non-sterile pharmaceutical products recalls from Acne cream, syrup, inhalation solution, non-alcohol body spray, baby oil, shampoo and mouthwashes. ‘*B. cepacia*’ contamination was associated with 34% of product recalls from 2004 to 2011 [6]. Examining FDA recalls from 2012 to 2019 [7] showed that *Burkholderia* spp. were the number one reason for non-sterile drug recalls (45.3%, 102 recalls), followed by *Ralstonia pickettii* (20%, 45 recalls) and *Salmonella* spp. (12.4%, 28 recalls). This led the United States Pharmacopeia (USP) to publish General Chapter (60) (*Microbiological examination of non-sterile products: Tests for Burkholderia cepacia complex*) (effective date: 1 December 2019) to detect the presence of BCC species in pharmaceutical substances and non-sterile pharmaceutical products [8]. The chapter provided methodology and testing parameters for the detection of species within the BCC. Recently, the FDA published a Pharmaceutical Microbiology Manual (PMM) to maximize the efficiency of our analytical results to support the Center for Drug Evaluation and Research (CDER) goal to assure the safety and reliability of commercially available medical products [9]. The PMM evolved from a sterility analytical manual and is a supplement to the USP (60), (61) and (62) for pharmaceutical microbiology testing.

USP methods for detecting bacteria include enrichment using Trypticase Soy Agar (TSA) or Trypticase Soy Broth (TSB) in traditional cultivation and phenotypic methods. Determination of total bacterial count is the basis for routine evaluation of microbiological quality in non-sterile pharmaceutical materials. One of our previous studies led to the development of robust BCC recovery using diluted growth media methods (i.e., 1/10 × TSA, 1/10 × TSB, Reasoner’s 2nd Agar (R2A) or Reasoner’s 2nd Broth (R2AB)), which allows improved recovery of BCC microorganisms that might be present in distilled water and preserved samples [10,11,12]. This research supports the utility of using diluted media and enriched cultures with a pre-enrichment step. The FDA made a recommendation to USP (60) for its adoption of the diluted growth media method as a compendial test method for BCC enumeration. However, these conventional methods, including enrichment using 1/10 × TSA or 1/10 × TSB, may be limited by their low sensitivity. Indeed, BCC strains were detected in 18.3 and 25.5% of tested samples from autoclaved nuclease-free water using 1/10 × TSA and 1/10 × TSB culture-based methods, respectively [12]. Furthermore, we demonstrated the potential of droplet digital polymerase chain reaction (ddPCR) and flow cytometry as more sensitive alternatives to culture-based methods to detect BCC in autoclaved nuclease-free water and antiseptic samples [12]. Given the aptitude of BCC to evade culture-based detection, their ability to grow in low-nutrient conditions, and their resistance to antimicrobial agents, and their inherent pathogenic potential, new detection methods can help to monitor the risk of BCC contamination and safety of a pharmaceutical product.

Rapid PCR and real-time quantitative PCR (qPCR) assays for the detection of BCC have been previously reported [3,12,13,14,15]. Mahenthiralingam et al. [16] used conventional PCR to detect BCC by means of amplification of its 16S ribosomal RNA (rRNA) gene and *recA* gene. The drawbacks of PCR and qPCR assays are characterized by both requiring expensive equipment and reagents, as well as proper training and technical expertise. The development of loop-mediated isothermal amplification (LAMP) was chosen over other amplification methods for three reasons, namely (i) LAMP can be performed by heating samples under an optimal temperature which ranges between 60–65 °C using a water bath or heat block, (ii) it can trace nucleic acids (1 pg/μL), and (iii) it can handle small amounts of reagents in a single reaction tube (up to a total volume of 10 μL) within 30 min [17,18]. The LAMP technique is based on the principle of strand displacement activity of *Bst* polymerase. It contains a set of six specially designed primers (two outer primers (F3 and B3), two inner primers (FIP and BIP), and two loop primers (LF and LB)), which recognize six different regions on the target nucleotide sequence. Positive and negative results can be discerned directly with the naked eye by adding phenol red, a well-known pH indicator [19,20]. Consequently, the reaction can be performed faster and at a much lower cost than by other PCR methods. Due to the high efficiency and sensitivity of LAMP, this method has been widely applied to pathogen detection [21,22,23,24,25,26].

Our aims in this study were as follows: First, to identify BCC-specific primers using whole genome sequence (WGS) data that enable differentiation of BCC from other *Burkholderia* species; Second, to develop LAMP assays for quantitatively assessing the specificity, and selectivity of these targets using the six robust primers; Third, to detect BCC from artificially inoculated distilled water deprived of any nutrient source, as well as from antiseptics (10 μg/mL chlorhexidine gluconate (CHX) or 50 μg/mL benzalkonium chloride (BZK)) for long-term incubation at 23 °C in the dark. If this approach proves feasible, this fast and easy-to-perform detection method for BCC could be easily used for testing non-sterile pharmaceutical drug and non-drug products.

## 2. Materials and Methods

### 2.1. LAMP Primer Design

All of the published complete genomes and genome metadata of the genus *Burkholderia*, including BCC, were retrieved from the PATRIC database (www.patricbrc.org/, accessed on 17 July 2021). In order to identify all possible BCC-specific PCR primers and amplicons, we used a pan-genome-based bioinformatics pipeline (Supplementary Figure S1): Step 1, identification of orthologous genes of *Burkholderia* genomes by cluster analysis (≥75% sequence identity) using UCLUST clustering algorithm in USEARCH; Step 2, generation of a GGM, weighted by the frequencies of pan-genome (i.e., the entire set of genes for the retrieved *Burkholderia* genomes); Step 3, identification of BCC-specific core gene(s) which are only present in BCC genomes from the GGM; Step 4, MSA analysis of DNA sequences of BCC-specific core gene(s); Step 5, identification of core DNA region(s) (i.e., possible BCC-specific PCR product regions including primer sequences) of the multiple sequence alignments; Step 6, in silico confirmation of the identified primers (and PCR product[s]) in terms of specificity and sensitivity using USEARCH BLAST against the genus *Burkholderia* genomes.

The specific LAMP primer sets developed and used in this study were designed using open-source software PrimerExplorer vs. 5 (http://primerexplorer.jp/lampv5e/index.html, accessed on 17 July 2021) (Table 1).

### 2.2. Bacterial Strains and DNA Preparation

Bacterial strains used in this research are listed in Table S1 in the Supplemental Material. These included 20 BCC strains, 18 non-BCC *Burkholderia* strains, and 18 non-*Burkholderia* strains. Thirty-eight strains of *Burkholderia* spp. were obtained from the *Burkholderia cepacia* Research Laboratory and Repository at the University of Michigan [10]. All BCC strains were cultivated as described previously [10], and non-BCC species and non-*Burkholderia* bacterial species were grown using TSA supplemented with 5% sheep blood (Blood Agar; Thermo Fisher Scientific, Waltham, MA, USA). Total genomic DNA was extracted from cultures of the tested strains using the DNeasy UltraClean Microbial Kit (Qiagen, Valencia, CA, USA) as directed by the manufacturer. Quality and quantity of the extracted genomic DNA were assessed using a NanoDrop ND-2000 spectrophotometer (Thermo Fisher Scientific Inc., Waltham, MA, USA) and normalized to either 1 or 2 ng/μL in nuclease-free water (pH 8.0; Thermo Fisher Scientific Inc., Waltham, MA, USA) for direct use in PCR.

### 2.3. LAMP and qPCR Assay

Assays were assembled in total reaction volumes of either 10 μL (for LAMP assays) or 20 μL (for qPCR). LAMP amplification was conducted with a final concentration of 10 ng/μL genomic DNA of *B. ambifaria* HI2468 as a template in a total volume of 10 μL containing 5 μL WarmStart Colorimetric LAMP 2× Master Mix (M1800L, New England Biolabs, Ipswich, MA, USA) and 1 μL of 10× primer mix containing 1.6 μM of each inner primer (FIP/BIP), 0.2 μM of each outer primer (F3, B3), and 0.4 μM of each loop primer (LF, LB) as recommended by the manufacturer (https://www.neb.com/protocols/2016/08/15/warmstart-colorimetric-lamp-2x-master-mix-typical-lamp-protocol-m1800, accessed on 17 July 2021). For a 96-well plate (evaluation of LAMP in autoclaved nuclease-free water, and antiseptic solutions), 100 reaction volumes (500 μL LAMP 2× Master Mix and 100 μL of 10× primer mix) were prepared and 6 μL were distributed to each well before adding 4 μL of genomic DNA samples. The T100^™^ Thermal Cycler (BioRad, Hercules, CA, USA) was used as a heat block to maintain a constant temperature of 65 °C for 25 min. The color was intensified by allowing the reaction to cool at 12 °C and the results were documented photographically.

qPCR amplification was carried out using 4 μL *B. ambifaria* HI2468 as a template in a total volume of 20 μL containing 10 μL 2× SsoAdvanced^™^ Universal SYBR^®^ Green Supermix (BioRad, Hercules, CA, USA), 0.6 μL each of 10 pM forward and reverse primers, and 4.8 μL ddH_2_O [12]. The PCR reaction was performed in a CFX96^™^ qPCR machine (BioRad, Hercules, CA, USA) with the following conditions: 98 °C for 2 min and 40 cycles of 98 °C for 5 s, 60 °C for 5 s, followed by 65–95 °C running for 10 s in each step in increments of 0.5 °C for the generation of a melting curve. For a 96-well plate (evaluation of qPCR in autoclaved nuclease-free water, and antiseptic solutions), 100 reaction volumes (1000 μL 2× SsoAdvanced^™^ Universal SYBR^®^ Green Supermix, 120 μL primer mix, and 480 μL ddH_2_O) were prepared and 16 μL were distributed to each well before adding 4 μL of genomic DNA samples. Thereafter, the qPCR and LAMP products were electrophoresed on 2% agar after GelRed^®^ Nucleic Acid Stain (C755G19, Thomas Scientific, Swedesboro, NJ, USA) staining and then detected under ultraviolet light in GelDoc Go Imaging System (BioRad, Hercules, CA, USA). Autoclaved nuclease-free water (Qiagen, Valencia, CA, USA) was used as the negative control in each run of the experiment.

### 2.4. Optimization of LAMP

Four reaction volumes of 5, 10, 15, and 20 μL; five temperatures of 55, 60, 65, 70, and 75 °C; and four time gradients of 15, 20, 25, and 30 min were successively conducted to determine the optimal LAMP conditions. The optimization procedure started with the four reaction volumes (i.e., 5, 10, 15, and 20 μL) of 10 ng/μL genomic DNA of *B. ambifaria* HI2468, which were incubated at 65 °C for 30 min. When one parameter was being optimized, the two other parameters were held constant. LAMP products originating from parameter optimization were analyzed using 2% agarose gel electrophoresis and visualized under a GelDoc Go Imaging System (BioRad, Hercules, CA, USA). This experiment was conducted in duplicate.

### 2.5. True-Positive Rate (Sensitivity) and True-Negative rate (Specificity) in Analysis Using LAMP

About 10 ng/μL genomic DNA of 56 bacterial strains were used as templates of the LAMP reaction to verify the optimal primer combination of BCC with non-target bacterial strains. The resulting LAMP products were analyzed with the naked eye directly and by 2% agarose gel electrophoresis following the procedures as described above. This experiment was conducted in duplicate. Sensitivity and specificity describe how well a test can determine whether a specific condition is present or absent [27]. The formulas used to calculate the estimated sensitivity and specificity are as follows:

Estimated sensitivity (%) = TP/(TP + FN) × 100

Estimated specificity (%) = TN/(FP + TN) × 100

(TP = number of true positive events, FP = number of false positive events, TN = number of true negative events, FN = number of false negative events)

Primer pairs exhibiting specific detection in LAMP were selected for further investigation and applicability in distilled water and antiseptics.

### 2.6. Limit of Detection (LOD) and Limit of Quantification (LOQ) of LAMP

The LOQ is the lowest analyte DNA concentration that provides an acceptable level of precision (i.e., 3/3 replicates amplified), whereas LOD is defined as the concentration of a measurand that is significantly different from a negative control (i.e., at least 2/3 replicates amplified) [28]. LOD and LOQ were measured through the qPCR and LAMP assays using tenfold serial dilutions of *B. cenocepacia* AU1054 and *B. cenocepacia* J2315 genomic DNA. LAMP products were directly analyzed with both the naked eye, and 2% agarose gel electrophoresis. Furthermore, the absorbance in LAMP products was measured using a Synergy MX spectrophotometer from BioTek Instruments, Inc. (Winooski, VT, USA) at 434 and 560 nm [19]. A reaction was considered positive when the color value was above 0.05 (ΔOD = OD_434nm_ − OD_560nm_; difference in absorbance of samples at 434 (increased absorbance) and 560 nm (decreased absorbance)). Negative reactions (i.e., wells with ΔOD values below 0.05) were analyzed by 2% agarose gel electrophoresis to confirm if a result is a true negative.

### 2.7. Evaluation of LAMP in Autoclaved Nuclease-Free Water and Antiseptic Solutions

#### 2.7.1. Autoclaved Nuclease-Free Water

BCC strains held in autoclaved nuclease-free water (Qiagen) were adjusted to a density corresponding to 0.08–0.1 absorbance at a wavelength of 600 nm (approximate cell density: 1.5 × 10^8^ colony-forming units (CFU/mL)) in the Synergy MX spectrophotometer (BioTek Instruments, Inc., Winooski, VT, USA) [11]. Each sample was prepared (23 January 2020) and stored at 23 °C (i.e., room temperature) for 361 consecutive days. Serial dilutions of each BCC suspension were prepared in 1 mL autoclaved nuclease-free water to yield appropriate CFUs (10, 10^2^, 10^3^, and 10^4^ CFU/mL). A total of 100 μL of each dilution (10, 10^2^, 10^3^, and 10^4^ CFU/mL) were boiled for 10 min in a water bath and were used as templates for qPCR and LAMP assays. Statistical analyses were performed using a one-way analysis of variance (one-way ANOVA), with a *p* value of <0.05 being considered significant.

#### 2.7.2. Chlorhexidine Gluconate (CHX) and Benzalkonium Chloride (BZK)

Autoclaved nuclease-free water samples containing BCC, as described previously, were treated with CHX and BZK, so that the final chemical concentrations reached 10 μg/mL for CHX and 50 μg/mL for BZK, respectively [11]. Each sample was prepared (September 8, 2020) and stored at 23 °C (at room temperature, in a dark environment such as in a closed laboratory cabinet) for 184 consecutive days. After that, sensitivity and specificity experiments were performed as described above using serial dilution equivalent to 10, 10^2^, 10^3^ and 10^4^ CFU/mL.

## 3. Results

### 3.1. Primers Designed for LAMP

We retrieved a total of 266 complete genomes (i.e., 82 of BCC and 184 of non-BCC, respectively) of the genus *Burkholderia*, together with their metadata, from the PATRIC database. From 814,642 gene sequences of the 266 *Burkholderia* genomes, our bioinformatics pipeline identified 174,715 clusters (i.e., orthologous gene groups with ≥75% sequence identity) and generated a genome-gene-matrix (GGM) (i.e., 266 × 174,715 matrix weighted by the frequencies of genes) (Supplementary Figure S1). Using GGM, we identified 206 BCC-specific clusters which are only present in BCC genomes. Among the 206 BCC-specific clusters, we chose a cluster, namely number of 2591 of ribB genes encoding a 3,4-dihydroxy-2-butanone 4-phosphate synthase (EC 4.1.99.12)/GTP cyclohydrolase II, which shows >92% sequence identity, and four completely conserved sequence regions (≥20 bp) for designing BCC-specific LAMP primers (Supplementary Table S2). We generated primer sets by PrimerExplorer vs. 5 (primerexplorer.jp/lampv5e/index.html, accessed on 17 July 2021), and evaluated three primer sets, namely RibB 5, RibB 16, and RibB 67 (Table 1 and Supplementary Figure S2).

### 3.2. Optimal Conditions of LAMP 

**Figure 1:**
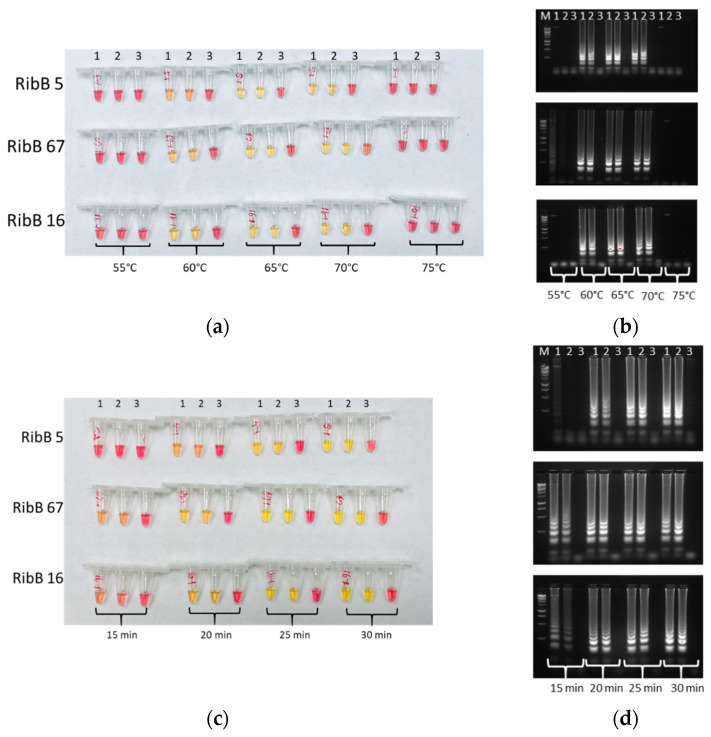
Different temperature and reaction time conditions of LAMP assay using 16.7 µg/mL and 1.67 µg/mL (20 µL reaction volume) of *B. ambifaria* HI2468. (**a**) Colorimetric LAMP for the detection of BCC at 55, 60, 65, 70 and 75 °C; (**b**) The LAMP reaction product (2.5 μL) was analyzed on a 2% agarose gel. (**c**) Colorimetric LAMP for the detection of BCC at 15, 20, 25, and 30 min using 16.7 µg/mL and 1.67 µg/mL of *B. ambifaria* HI2468 at 65 °C.; (**d**) Results were confirmed by gel electrophoresis. Top row, RibB 5 primer; middle row, RibB 67 primer and bottom row, RibB 16 primer. M: 100 bp DNA ladder. 1: 16.7 µg/mL, 2: 1.67 µg/mL of *B. ambifaria* HI2468. 3: negative control-nuclease free water.

The optimal conditions of the primer combination RibB 5, RibB 16, and RibB 67 were examined by LAMP assays. Several critical variables, including reaction volume, temperature, and time, were optimized for the LAMP reaction. To optimize incubation temperatures, initial LAMP incubated at 55, 60, 65, 70 and 75 °C for 30 min on 16.7 ng/μL genomic DNA of *B. ambifaria* HI2468. LAMP products obtained were obviously different between below 60 and above 75 °C, which means 65 °C can be considered the optimal reaction temperature (Figure 1). Gel electrophoresis revealed distinct banding patterns for LAMP products (Figure 1b). Optimal incubation time for colorimetric LAMP at 65 °C on 16.7 ng/μL genomic DNA is 25 min. LAMP products analyzed by color and agarose gel electrophoresis remained relatively unchanged over time from 25 to 30 min (Figure 1). The shortest possible reaction time is 15 min with slightly different color and gel electrophoresis band intensity. Four reaction volume gradients of 5, 10, 15, and 20 μL at 65 °C for 25 min on 10 ng/μL genomic DNA samples changed color (Supplementary Figure S2). This finding indicates that 5 μL was the minimum reaction volume. Considering detection efficiency, sensitivity and further analysis, a volume of 10 μL was selected. Thus, the optimal LAMP reaction parameters were 10 μL, 65 °C, and 25 min, which were applied throughout the rest of this study.

### 3.3. Specificity Analysis of LAMP

**Figure 2:**
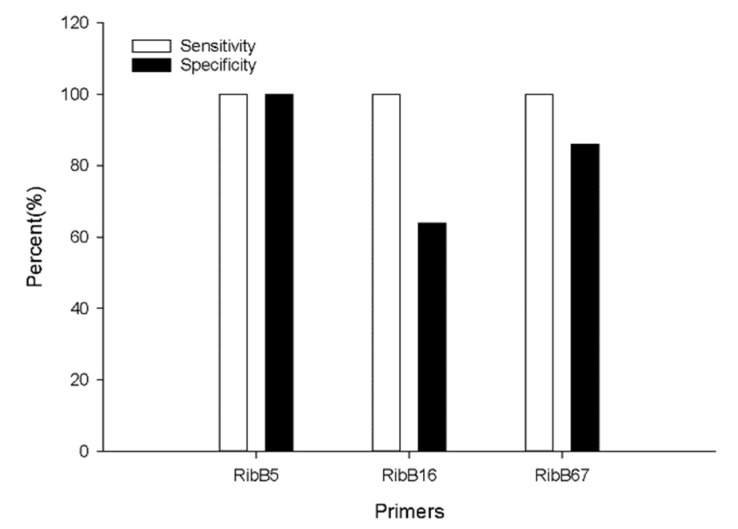
Comparison of sensitivity and specificity of three designed primers for the LAMP assay. Sensitivity of RibB 5 = TP/(TP + FN) × 100 = 20/(20 + 0) × 100 = 100%; Specificity of RibB 5 = (TN/(FP + TN) × 100 = 36/(0 + 36) × 100 = 100%.

To investigate the specificity of colorimetric LAMP assay with the newly designed RibB 5, RibB 16, and RibB 67 primers, genomic DNA of 56 bacterial strains (i.e., 38 *Burkholderia* strains (20 BCC strains and 18 non-BCC strains) and 18 non-*Burkholderia* bacterial species) were extracted individually and about 10 ng/μL were used for the LAMP reaction. As shown in Table S1 in the Supplemental Material, LAMP assays with all primers were positive when the genomic DNA derived from 20 BCC strains were used as templates of the LAMP reaction ((Number of true positive events (TP)/(TP + Number of false negative events (FN)) × 100 = 20/(20 + 0) × 100 = 100% sensitivity; 20 positives out of 20). The RibB 5 primers did not change colors with 18 non-BCC strains and 18 non-*Burkholderia* bacterial species. The RibB 16 primers did not change color with 18 non-*Burkholderia* strains whereas color change was observed in the following 6 strains out of 18 non-BCC: *B. concitans* AU12121, *B. fungorum* AU18377, *B. gladioli* AU26454, *B. gladioli* AU30473, *B. tropica* AU19944, and *Caballeronia zhejiangensis* AU12096. The RibB 67 primers amplified the 6 strains out of 18 non-Burkholderia strains and 7 strains out of 18 non-BCC strains, respectively. Based on these results, the specificity of RibB 5, RibB 16, and RibB 67 were 100% ((Number of true negative events (TN)/(Number of false positive events (FP) + TN) × 100 = 36/(0 + 36) × 100 = 100%; 36 negative of 36), 63.9% (23 negative out of 36) and 86.1% (31 negative out of 36), respectively (Figure 2). On the basis of these results, we decided to use the RibB 5 primer set for follow-up experiments and to compare our results with qPCR performed.

### 3.4. Limit of Quantification (LOQ) and Limit of Detection (LOD) of the LAMP Assay 

**Figure 3:**
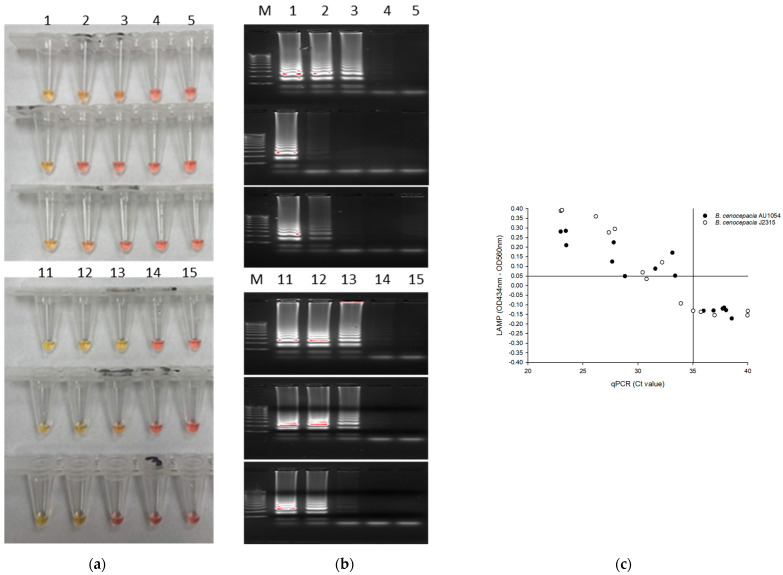
Limit of detection (LOD) of LAMP using 10-fold serial dilutions of *B. cenocepacia* AU1054 and *B. cenocepacia* J2315 ranging from 100 pg/µL to 10 fg/µL. (**a**) Colorimetric LAMP for the detection of BCC using RibB 5 primer; (**b**) Results were confirmed by gel electrophoresis; (**c**) Scatter plot of ΔOD values (OD_434nm_–OD_560nm_) versus C_T_ values. M; Maker, 1: 103.4 pg/µL, 2: 10.34 pg/µL, 3: 1.034 pg/µL, 4: 103.4 fg/µL, 5: 10.34 fg/µL of *B. cenocepacia* AU1054, 11: 111.7 pg/µL, 12: 11.17 pg/µL, 13: 1.117 pg/µL, 14: 111.17 fg/µL, 15: 11.17 fg/µL of *B. cenocepacia* J2315.

To determine the LOQ and LOD of the qPCR and LAMP assay, we tested the RibB 5 primers sets using diluted series of genomic DNA of *B. cenocepacia* AU1054 (103.4 pg/μL, 10.34 pg/μL, 1.034 pg/μL, 103.4 fg/μL, and 10.34 fg/μL) and *B. cenocepacia* J2315 (111.7 pg/μL, 11.17 pg/μL, 1.117 pg/μL, 111.7 fg/μL, and 11.17 fg/μL). Figure 3a shows that the oligonucleotide set for the RibB gene was capable of detecting 1 pg/μL DNA molecules in a test reaction, as evidenced by the red-to-yellow color change. Analysis by gel electrophoresis revealed clearly distinct banding patterns for the LAMP reaction products (lanes with ≥1 pg/μL) (Figure 3b). These results indicated that the LOQ and LOD of the LAMP assay with the RibB 5 primers was 10.34 pg/μL and 1.034 pg/μL, equivalent to 0.22 ± 0.12 and 0.089 ± 0.05 color values, respectively. On the basis of this 1 pg/μL observation, we considered the ΔOD > 0.05 value as positive.

The qPCR reaction with the RibB 5 primers corresponds to a 32.04 ± 0.98 and 30.88 ± 1.11 cycle threshold values (C_T_) per 1.034 pg/μL and 1.117 pg/μL of *B. cenocepacia* AU1054 and J12315. Furthermore, about 100 fg/μL of AU1054 were amplified and emerged on the gel, corresponding to a value of 34.49 ± 0.39 C_T_. On the basis of gel electrophoresis, we selected a C_T_ ≤ 35 value as a positive. Plotting ΔOD values versus C_T_ values of qPCR revealed that samples with a C_T_ ≤ 35 showed a red to yellow color change in the LAMP test (Figure 3c). Moreover, 6 samples with C_T_ values between 30 and 35 showed a color change, while 3 other samples in the same range did not. The LOD of qPCR was 35 C_T_, equivalent to 103.4 fg/μL and 111.7 fg/μL for *B. cenocepacia* AU1054 and *B. cenocepacia* J2315, respectively. Thus, qPCR showed a tenfold greater sensitivity than LAMP for both strains.

### 3.5. Testing BCC Detection from Autoclaved Nuclease-Free Water, and Antiseptic Solutions with the LAMP Assay

**Figure 4:**
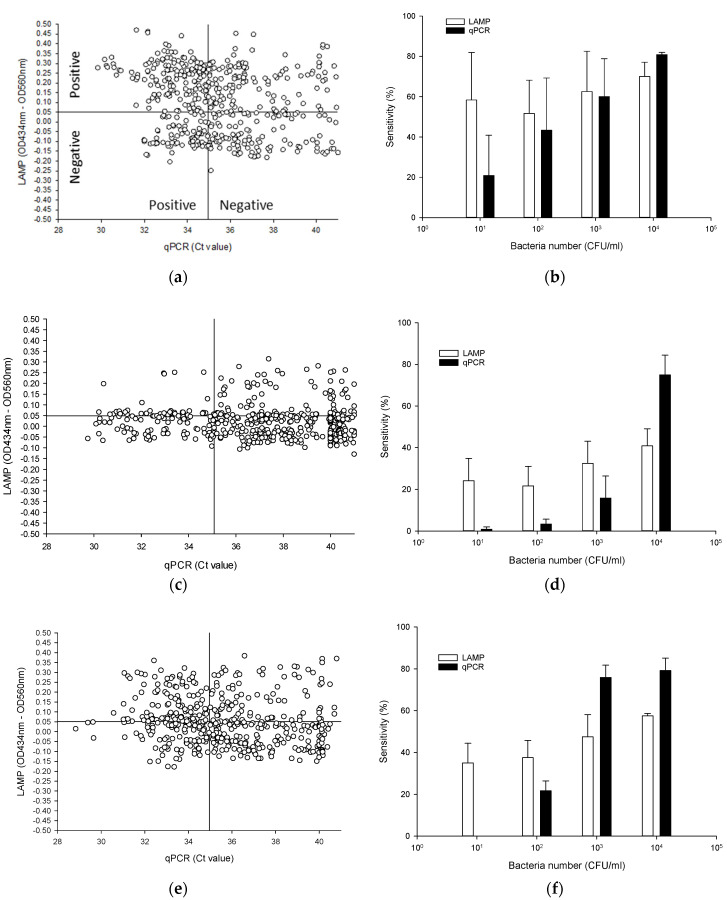
Detection of BCC using LAMP with RibB 5 primer from nuclease-free distilled water samples (**a**,**b**), 10 μg/mL CHX solution (**c**,**d**) and 50 μg/mL BZK solution (**e**,**f**). (**a**,**c**,**e**) Scatter plot of ΔOD values (OD_434nm_–OD_560nm_) versus C_T_ values. (**b**,**d**,**f**) Comparison of LAMP and qPCR sensitivity for positive BCC detection from BZK.

To evaluate the colorimetric LAMP assay, we compared its sensitivity to a validated qPCR method. We first used 480 samples (20 strains × 4 serial dilutions × 6 replicates = 480 samples) and performed LAMP reactions using 2 μL of isolated DNA in a reaction volume of 10 μL. Genomic DNA of bacteria at inoculation levels of 10, 10^2^, 10^3^ and 10^4^ CFU/mL determined via plate counting, was boiled in water to extract DNA that was then detected by qPCR and LAMP. To visualize these data, we plotted ΔOD values against C_T_ values obtained from the qPCR method in autoclaved nuclease-free water, and antiseptic solutions. To determine the sensitivity of the LAMP assay, we used ΔOD values > 0.05 and counted them as “TP”. Likewise, OD values < 0.05 were counted as “FN”. qPCR sensitivity used the C_T_ ≤ 35 value (TP) and C_T_ > 35 value (FN). (Figure 4; see also Supplementary Tables S3–S5).

#### 3.5.1. Nuclease-Free Water

A comparison of the sensitivity of the qPCR and LAMP methods used to detect BCC strains from autoclaved nuclease-free water at 23 °C over 361 days is presented in Figure 4 (Supplementary Table S3). Among the 480 tests, 291 were TP for LAMP (ΔOD > 0.05), while the remaining 189 tests were FN. TP results for qPCR was observed in tests with C_T_ values above 35. qPCR revealed 246 TP tests (C_T_ ≤ 35), and 234 FN tests (Figure 4a). The sensitivity percentages of tests done with autoclaved nuclease-free water through qPCR method (51.3%; TP/(TP + FN) × 100 = 246/(246 + 234) × 100) were significantly lower (*p* < 0.05) than those obtained through LAMP (60.6%; TP/(TP + FN) × 100 = 291/(291 + 189) × 100).

LAMP and qPCR assay—using all serial dilution inoculation methods—could detect ≥10 CFU/mL of BCC from autoclaved nuclease-free water samples (Figure 4b, Supplementary Table S3). LAMP and qPCR assay provided high detection capabilities, showing sensitivity above 62/120 (51.7%) except for the qPCR assay (25/120 (20.8%) and 52/120 (43.3%)) at 10 and 10^2^ CFU/mL samples. Although the LAMP method showed slightly higher sensitivity compared to the qPCR method at 10^2^ and 10^3^ CFU/mL samples, there was no significant difference (*p* > 0.05) as far as sensitivity was concerned. Among the 480 samples, 2 samples (i.e., *B. cepacia* AU24442 in 10^2^ CFU/mL and *B. cepacia* PC783 in 10^4^ CFU/mL) were not amplified during one of the six tests for LAMP. Meanwhile, the qPCR assay (97/120; 80.8%) appears to yield a higher detection capability compared to LAMP for BCC at 10^4^ CFU/mL samples.

#### 3.5.2. CHX

The number of TP tests for 10 μg/mL CHX at 23 °C over 184 days with the LAMP and qPCR methods were 114 (out of 480; 23.8%) and 143 (out of 480; 29.8%), respectively (Figure 4c) (Supplementary Table S4). Most ΔOD values of BCC-positive samples for CHX were close to 0.05, which was observed in tests where C_T_ values were above 35.

In CHX samples, qPCR and LAMP could also detect ≥10 CFU/mL of BCC (Figure 4d) (Supplementary Table S4). The sensitivity percentages in total CHX samples obtained using the LAMP (71.3%) were significantly higher (*p* = 0.0001) than those obtained using qPCR method (40%). Among the 240 samples at 10 and 10^2^ CFU/mL samples, 29 and 26 were TP for LAMP, while the remaining 91 and 94 samples were FN. qPCR revealed 1 and 4 TP samples, and 119 and 116 FN samples at 10 and 10^2^ CFU/mL samples. The sensitivity was significantly different (*p* < 0.05) between LAMP (24.2%, 21.7% and 32.5%) and qPCR methods (0.83%, 3.3% and 15.8%) in 10 to 10^3^ CFU/mL dilution samples. However, in 10^4^ CFU/mL dilution samples, the sensitivity of LAMP (40.8%) was significantly lower (*p* < 0.05) than that of the qPCR (75%) method.

#### 3.5.3. BZK

The TP in total 50 μg/mL BZK samples at 23 °C over 184 days obtained using the qPCR method (213 out of 480; 44.4%) were similar to those obtained using LAMP (211 out of 480; 43.9%) (Figure 4e) (Supplementary Table S5). As in autoclaved nuclease-free water and in CHX, TP results for LAMP in BZK were observed in qPCR tests with C_T_ > 35.

The sensitivity percentage of the LAMP method was above 35% in all serial dilution samples (Figure 4f). No amplification by qPCR was observed when the target concentration was 10 CFU/mL samples. The detection limit of LAMP was 10 CFU/mL, and that of qPCR was 10^2^ CFU/mL. Interestingly, in low dilution samples (10 and 10^2^ CFU/mL), the sensitivity percentage of LAMP (35% and 37.5%) was significantly lower (*p* < 0.05) than those of qPCR (0% and 21.7%). However, in 10^3^ and 10^4^ CFU/mL dilution samples from nuclease free water, the sensitivity of LAMP (47.5% and 57.5%) was significantly lower (*p* < 0.05) than that of qPCR (75.8% and 78.3%) (Figure 4f) (Supplementary Table S5).

## 4. Discussion

BCC contamination of pharmaceutical products is a major concern. Recalls in the past few years revealed that the most commonly detected microorganisms found in aqueous formulations were *Burkholderia* spp., *Ralstonia pickettii* and *Salmonella* spp. [7,29]. Therefore, rapid, economical, and executable methods in low-resource laboratory settings for the detection of BCC are required. The *ribB*-based colorimetric LAMP assay described here can be performed in 10 μL final volume within 25 min at 65 °C. We observed that after optimization, this assay was able to detect BCC in nuclease-free water after 361 days, in 10 μg/mL CHX solutions after 184 days, and in 50 μg/mL BZK solutions after 184 days.

Typically, four primer sequences of LAMP assay are sufficient to amplify a target nucleic acid [18]. However, in order to enhance the specificity and efficacy of the reaction, two additional loop primers (e.g., Loop F and Loop B) were incorporated in the LAMP reaction mixture [18]. The difficulty of the LAMP assay resides in the complexity of primer design due to the number of primers in the LAMP primer set [18,19,21]. In addition, considering the close genomic and phenomic relatedness of BCC with other *Burkholderia* strains, it is also obvious that the successful design of BCC-specific LAMP primers chiefly depends on finding BCC-specific target sequence(s) with sufficiently conserved common regions as annealing sites for the LAMP primers. For these reasons, we adopted a pan-genome based approach using all available *burkholderia* complete genome sequence data to obtain a Venn diagram output of the genes from two groups (i.e., BCC group and non-BCC group) (Supplementary Figure S1a). The set of genes in the core-genome of BCC but not belonging to the pan-genome of non-BCC species (i.e., the relative complement of the pan-genome of non-BCC in the core-genome of BCC) provided a database of candidate genes with BCC-specificity for subsequent searching the most promising BCC-specific target gene(s). Interestingly, among >174,000 orthologous gene clusters (with ≥75% sequence identity) observed across all 266 *Burkholderia* strains, we found 206 BCC-specific gene clusters. These could be used for all PCR-based detection methods for BCC and also provide a genome-centric insight for the distinguishable phenotypic feature(s), accurate identification, and epidemiological/environmental characterization of BCC [1,30]. Hybridization of BCC-specific primers to the target DNA is a crucial step for the efficiency of LAMP. Among the 206 candidate clusters, we chose a cluster of *ribB* genes encoding a 3,4-dihydroxy-2-butanone 4-phosphate synthase (EC 4.1.99.12)/GTP cyclohydrolase II by using detailed analysis of conserved regions in the multiple sequence alignment (MSA). The *ribB* genes with > 92% sequence identity have four completely conserved sequence regions (≥20 bp) with no cross homology, which satisfy the requirements for designing BCC-specific LAMP primers. The specificity and sensitivity of the *ribB*-based BCC-specific LAMP primers were verified by both BLAST analysis and full-scale lab experiments.

The LAMP assay with RibB 5 primer exhibits a much lower limit of detection 1 pg/μL compared to the 22 ng of *B. mallei* ATCC 325 reported by Mirzai et al. [24]. Similarly, the LAMP assay reported by Pal et al. [31] could detect as low as 1 pg of *B. mallei* NCTC 10230. Saxena et al. [26] has determined *B. mallei* NCTC 10245 using as little as by 250 fg of genomic DNA. Furthermore, the LAMP assay could detect 4.73 × 10^2^–2.1 × 10^3^ CFU/mL *B. mallei* NCTC 10245 of artificially spiked tap water and human blood [26]. In this study, the LAMP assay using 10 CFU/mL inocula provided high detection capability, showing sensitivity of 58.3% (70/120, 70 positives out of 120 samples) in autoclaved nuclease-free water, 24.2% (29/120) in CHX, and 35% (42/120) in BZK. These results were 10-fold more sensitive than semi-nested PCR (SN-PCR), which has a detection limit of 10^2^ CFU/mL for *B. cepacia* (70%, 7/10) [13]. However, conventional qPCR has been reported to exhibit higher sensitivities, detecting as low as 10 fg/μL of *B. cepacia* DNA [15], which is a 10-fold more sensitive than the qPCR assays we have been using thus far [12]. Although qPCR is highly sensitive, analyzed samples required about 30 h, which included enrichment (24 hrs), DNA extraction (30 min), and qPCR reactions (3 h) [12,15]. Many of the samples currently analyzed by USP (60) (microbiological examination of nonsterile products—Tests for *Burkholderia cepacia* complex) were plated on *Burkholderia Cepacia* Selective Agar (BCSA; Remel, Lenexa, KS, USA) [8], which required a median time of 16.5 days for the first positive culture from contaminated liquid docusate [29]. In this study, samples were boiled in a water bath for 10 min and were used as a template DNA for LAMP assays without any purification or precipitation. Simply boiling BCC strains in autoclaved nuclease-free water and antiseptics to produce a template was successful for the direct amplification of BCC strains [12]. The LAMP assay requires about 1 h without any enrichment, thus constituting a sensitive, rapid, cost-effective and simple assay.

Although LOD of LAMP was a 10-fold lower than qPCR, in 10 CFU/mL dilution samples, the sensitivity percentage of LAMP (58.3% (70/120), 24.2% (29/120), and 35% (42/120)) was significantly higher (*p* < 0.05) than those of the qPCR (20.8% (25/120), 0.8% (1/120), and 0% (0/120)) method from autoclaved nuclease-free water, CHX and BZK. These sensitivity percentages did not overlap the detection limit between LAMP and qPCR. These different results between the detection limit and sensitivity percentage can be explained by false-positive amplification products. LAMP relies on 6 different primers, suggesting a higher degree of specificity than conventional PCR and qPCR using two primers [17,18]. This makes LAMP assay not only susceptible to the formation of primer dimers, but also cross contamination by aerosol leading to false-positive results much easier than conventional PCR and qPCR [19,32,33,34]. Indeed, in this study, we have observed some qPCR negative samples (< 100 fg/μL) with C_T_ values between 35 to 40, which triggered color changes that were caused by non-specific amplification products (data not shown). These negative qPCR samples should not trigger a color change in LAMP, due to the detection limit of the LAMP assay (ΔOD < 0.05) of 1 pg/μL of BCC genomic DNA. This was more likely the result of false-positive amplification products, which is a well-known problem with LAMP [33]. Dao et al. [19] were successful in identifying the false-positive samples in the colorimetric LAMP assay with PCR using the combination of gel electrophoresis and internal standard. In the current study, to prevent cross-contamination, we took several precautions, including aseptic cleaning carried out before and after LAMP assays, 10× primer mix was prepared for 100 reactions for a 96-well plate, and plates were sealed to shield from aerosols. Furthermore, to eliminates false-positive results, even though the procedure is more time-consuming, we recommend the pre-enrichment step of product suspension to reach a level above 10^4^ CFU/mL, which allows for improved sensitivity of the LAMP assay of samples from distilled water and antiseptic samples. For instance, in 10^4^ CFU/mL dilution samples, bacteria were recovered with a greater efficiency through LAMP (70% (84 out of 120), and qPCR (C_T_ ≤ 35; 80.8% (97/120)) from autoclaved nuclease-free water. These results were consistent with recently severe acute respiratory syndrome coronavirus-2 (SARS-CoV-2) studies in that they showed good sensitivity for samples up to C_T_ < 30 [19]. Overall, to reduce the false-positive amplicon, we strongly recommend LAMP samples be handled with extreme caution, including examining repeatability (intraassay variance) and reproducibility (intraassay variance).

LAMP has previously been used to detect *B. mallei* and *B. pseudomallei* using turbidity and SYBR Green I in the reaction mix [23,31]. WarmStart master mix in LAMP reactions can simply be visualized by adding pH sensitive dyes such as phenol red [20]. Phenol red is a pH indicator which turns yellow below a pH of 6.4 and red above a pH of 8.2. If the DNA polymerase incorporates a deoxynucleotide triphosphate into the nascent DNA, the pyrophosphate moiety and a hydrogen ion are released, which turn the solution color from pink to yellow [20,35]. A pH sensitive dye added to the reaction mixture resulted in a color change from red to yellow in a positive test and remained red in negative tests. Alternatively, by using the Synergy MX spectrophotometer (BioTek Instruments, Inc., Winooski, VT, USA) at 434 and 560 nm, it was more effective to determine a positive reaction (ΔOD values > 0.05), compared to assessment with the naked eye or by gel electrophoresis. However, colorimetric LAMP appears to be running reactions in a weakly buffered environment [20]. Some non-sterilized pharmaceutical products contain contaminants that can lead to acidification or alkalization of the reaction environment, independent of the presence of a BCC template. Lin et al., [36] reported that the pH value of 0.02% CHX in sterile water was 5.9. In this study, the 0.001% (10 μg/mL) CHX and 0.005% (50 μg/mL) BZK without BCC has not triggered a color change from red to yellow. However, the number of TP samples for CHX with LAMP method was 143 (out of 480 tests), lower than 291 from autoclaved nuclease-free water and 213 from BZK. Therefore, CHX might have an effect on pH buffering to phenol red, but demonstrating this hypothesis requires further study.

Although the LAMP assay has shown to detect *B. mallei* and *B. pseudomallei* despite limitations [24,26,31], it depended on the type of various non-sterilized pharmaceutical products. Based on the simplest methods to get the shortest procedure, including DNA extraction by boiling procedure, the cost of LAMP per test is also considerably lower than other available molecular tests [37]. Such a small reaction volume can greatly reduce the cost of LAMP applications and may gain wider acceptance in testing the safety of pharmaceutical products. The cost-efficiency (250 reactions (10 μL) = about USD 250; approx. USD 1/reaction) of LAMP assays makes it acceptable to use even in resource-limited laboratories of smaller pharmaceutical manufacturers [38]. Furthermore, LAMP can be carried out by trained undergraduate students in an unclassified environment, without the need for specialized equipment and/or expensive supplies [38].

## 5. Conclusions

Non-sterile water-based drug and non-drug products have been shown to be contaminated with the BCC and have caused pharmaceutical product recalls within the U.S. A simple and rapid detection of BCC in non-sterile pharmaceutical products is critical to ensure consumer safety. In this study, we evaluated a colorimetric LAMPto detect BCC in autoclaved nuclease-free water, 10 μg/mL CHX solutions and 50 μg/mL BZK solutions. According to results from LAMP and qPCR used in this study, LAMP tests proved to be less technically demanding, faster, and cost effective. Thus, LAMP assays, combined with DNA extraction by boiling, can be used as effective methods to detect BCC strains found in drug ingredients, pharmaceutical-grade water, and finished pharmaceutical products.

## Figures and Tables

**Table 1:** Primer sequences for qPCR and loop-mediated isothermal amplification assay (LAMP).

	Primers		Sequences (5′-3′)	Length (bp)
	qPCR	LAMP		
RibB5	RibB5-F	RibB5 F3	GGCCGGATGGTGATCCT	17
	RibB5-R	RibB5 B3	GTCATCAGCGGCAGGTG	17
		RibB5 FIP	TCCGGTGTGACGAATTCGGC- CGAAGAAGACCGCGAAAACG	40
		RibB5 BIP	AACTTCATGGCCAAGTACGGCC-AAGCTGCTTGCAGCGTT	39
		RibB5 LF	GATCACGAGGTCGCCCT	17
		RibB5 LB	CGGCCTGGTTTGTCTGA	17
RibB67	RibB67-F	RibB67 F3	GCGAYACGAAGGAACAYCTG	20
	RibB67-R	RibB67 B3	CGTAGCCGGACATGCTG	17
		RibB67 FIP	TGAAATCGACCGGCCGGC-TCTTCAAGGCGTTCGACGAG	38
		RibB67 BIP	AGATCCTGCGCGATGTCGG-CCAGCTTGCGCGGATTC	36
		RibB67 LF	TGAGCGCAGCGGCCTTTTCTT	21
		RibB67 LB	TCGGCAAGATGCAGGTGC	18
RibB16	RibB16-F	RibB16 F3	ARGGCGACCTCGTGATC	17
	RibB16-R	RibB16 B3	CGAGATGCCGGTCGTCAC	18
		RibB16 FIP	TCAGACAAAYCAGGCCGCG-CGTCACACCGGAAGCGAT	37
		RibB16 BIP	ACGCTGCAAGCAGCTTCACC-CGATGCTGACCGTGAACG	38
		RibB16 LF	CCGTACTTGGCCATGAAGTT	20
		RibB16 LB	GCTGATGACCTACCGCAAC	19

Abbreviations: F, Forward Primer; R, Reverse Primer; F3, Forward Outer Primer; B3, Backward Outer Primer; FIP, Forward Internal Primer; BIP, Backward Internal Primer; LF, Loop Forward Primer; LB, Loop Backward Primer Y = T/C, R = G/A.

## Data Availability

Not applicable.

## Supplementary Materials

The following are available online at https://www.mdpi.com/article/10.3390/pathogens10091071/s1, Figure S1: Venn diagram for the clusters of BCC group and non-BCC group. Among 174,715 orthologous gene clusters (with ≥75% sequence identity) observed across all 266 complete *Burkholderia* genomes, 206 BCC-specific gene clusters were identified (a) and work flow of primer design process (b). Figure S2: Different reaction volume conditions (5, 10, 15, and 20 µL) of LAMP assay using 10.3 µg/mL and 1.03 µg/mL of *B. cenocepacia* AU1054 at 65 °C. Colorimetric LAMP for the detection of BCC using RibB 16 primer. Table S1: Specificity analysis of LAMP for BCC. Table S2: BCC cluster multiple sequence alignment (MSA)_summary. Table S3: Comparison of positive results for *B. cepacia* complex using LAMP and qPCR in nuclease-free water. Table S4: Comparison of positive results for *B. cepacia* complex using LAMP and qPCR in CHX. Table S5: Comparison of positive results for *B. cepacia* complex using LAMP and qPCR in BZK.
