# Supplementary material for: Loop-Mediated Isothermal Amplification (LAMP) Assay for Detecting Burkholderia cepacia Complex in Non-Sterile Pharmaceutical Products
Source: Pathogens. 2021 Aug 24;10(9):1071. doi: 10.3390/pathogens10091071 (PMC8468478; doi:10.3390/pathogens10091071)
Supplement: Supplementary file 1 [file pathogens-10-01071-s001.zip › Supp-LAMP for detecting Burkholderia cepacia complex.pdf]

**Supplementary Material**

Table S1. Specificity analysis of LAMP for BCC.

| | No | Strain name | Primers | | |
|---|---|---|---|---|---|
| | | | RibB 5 | RibB 16 | RibB 67 |
| Non-*Burkholderia* | *1* | *Enterococcus faecalis* ATCC 29212 | - | - | - |
| | *2* | *Enterococcus durans* ATCC 6056 | - | - | - |
| | *3* | *Proteus mirabilis* ATCC 7002 | - | - | - |
| | *4* | *Enterococcus faecium* ATCC 35667 | - | - | - |
| | *5* | *Bacillus subtilis* ATCC 6051 | - | - | - |
| | *6* | *Citrobacter freundii* ATCC 8090 | - | - | - |
| | *7* | *Pseudomonas aeruginosa* PAO1 | - | - | - |
| | *8* | *Enterococcus faecium* ATCC 49624 | - | + | - |
| | *9* | *Yersinia enterocolitica* subsp. enterocolitica ATCC 27729 | - | - | - |
| | *10* | *Shigella sonnei* ATCC 9290 | - | - | - |
| | *11* | *Lactobacillus salivarius* subsp. salivarius ATCC 11741 | - | + | - |
| | *12* | *Enterobacter aerogenes* ATCC 13048 | - | - | - |
| | *13* | *Klebsiella pneumoniae* ATCC 13883 | - | - | - |
| | *14* | *Pseudomonas aeruginosa* ATCC 27853 | - | + | - |
| | *15* | *Candida albicans* (Robin) Berkhout ATCC 10231 | - | - | - |
| | *16* | *Salmonella enterica* | - | + | - |
| | *17* | *Paenibacillus lautus* | - | + | - |
| | *18* | *Brevibacillus laterosporus* | - | + | - |
| BCC | *19* | *Burkholderia cepacia* PC783 | + | + | + |
| | *20* | *Burkholderia cepacia* AU24442 | + | + | + |
| | *21* | *Burkholderia stabilis* AU23340 | + | + | + |
| | *22* | *Burkholderia pyrrocinia* AU11057 | + | + | + |
| | *23* | *Burkholderia ambifaria* HI2468 | + | + | + |
| | *24* | *Burkholderia anthina* HI2738 | + | + | + |
| | *25* | *Burkholderia metallica* AU0553 | + | + | + |
| | *26* | *Burkholderia metallica* AU16697 | + | + | + |
| | *27* | *Burkholderia contaminans* HI3429 | + | + | + |
| | *28* | *Burkholderia contaminans* AU24637 | + | + | + |
| | *29* | *Burkholderia diffusa* AU1075 | + | + | + |
| | *30* | *Burkholderia arboris* ES0263a | + | + | + |
| | *31* | *Burkholderia arboris* AU22095 | + | + | + |
| | *32* | *Burkholderia lata* HI4002 | + | + | + |
| | *33* | *Burkholderia cenocepacia* AU1054 | + | + | + |
| | *34* | *Burkholderia cenocepacia* AU0222 | + | + | + |
| | *35* | *Burkholderia cenocepacia* AU19236 | + | + | + |
| | *36* | *Burkholderia cenocepacia* HI2976 | + | + | + |
| | *37* | *Burkholderia cenocepacia* HI2485 | + | + | + |
| | *38* | *Burkholderia cenocepacia* J2315 | + | + | + |
| Non-BCC | *39* | *Burkholderia concitans* AU12121 | - | - | + |
| | *40* | *Burkholderia fungorum* AU18377 | - | + | +- |
| | *41* | *Burkholderia fungorum* AU35949 | - | + | - |
| | *42* | *Burkholderia gladioli* AU16341 | - | - | - |
| | *43* | *Burkholderia gladioli* AU26454 | - | - | +- |
| | *44* | *Burkholderia gladioli* AU29541 | - | - | - |
| | *45* | *Burkholderia gladioli* AU30473 | - | - | +- |
| | *46* | *Burkholderia glumae* AU6208 | - | - | - |
| | *47* | *Burkholderia glumae* AU12450 | - | + | - |
| | *48* | *Burkholderia oklahomensis* ES0634 | - | - | - |
| | *49* | *Burkholderia plantarii* AU9801 | - | - | - |
| | *50* | *Burkholderia plantarii* AU37486 | - | + | - |
| | *51* | *Burkholderia thailandensis* AU13555 | - | + | - |
| | *52* | *Burkholderia thailandensis* AU36262 | - | + | - |
| | *53* | *Burkholderia tropica* AU15822 | - | - | - |
| | *54* | *Burkholderia tropica* AU19944 | - | + | + |
| | *55* | *Caballeronia zhejiangensis* AU10475 | - | - | - |
| | *56* | *Caballeronia zhejiangensis* AU12096 | - | - | + |

Table S3. Comparison of positive results for *B. cepacia* complex using LAMP and qPCR in nuclease-free water.

| | | Inoculum (CFU/ml) | | | | | | | |
|---|---|---|---|---|---|---|---|---|---|
| | | 10 | | $10^2$ | | $10^3$ | | $10^4$ | |
| | | LAMP | qPCR | LAMP | qPCR | LAMP | qPCR | LAMP | qPCR |
| 1 | *Burkholderia cepacia* PC783 | 2/6[a] | 0/6 | 2/6 | 1/6 | 2/6 | 2/6 | 0/6 | 3/6 |
| 2 | *Burkholderia cepacia* AU24442 | 5/6 | 1/6 | 0/6 | 2/6 | 2/6 | 3/6 | 3/6 | 3/6 |
| 3 | *Burkholderia stabilis* AU23340 | 2/6 | 3/6 | 1/6 | 3/6 | 2/6 | 4/6 | 2/6 | 6/6 |
| 4 | *Burkholderia pyrrocinia* AU11057 | 1/6 | 1/6 | 1/6 | 1/6 | 1/6 | 4/6 | 1/6 | 6/6 |
| 5 | *Burkholderia ambifaria* HI2468 | 2/6 | 0/6 | 1/6 | 4/6 | 3/6 | 6/6 | 3/6 | 6/6 |
| 6 | *Burkholderia anthina* HI2738 | 2/6 | 2/6 | 3/6 | 2/6 | 2/6 | 3/6 | 2/6 | 6/6 |
| 7 | *Burkholderia metallica* AU0553 | 6/6 | 4/6 | 6/6 | 6/6 | 5/6 | 6/6 | 6/6 | 6/6 |
| 8 | *Burkholderia metallica* AU16697 | 5/6 | 5/6 | 6/6 | 6/6 | 3/6 | 4/6 | 6/6 | 6/6 |
| 9 | *Burkholderia contaminans* HI3429 | 6/6 | 0/6 | 5/6 | 4/6 | 5/6 | 3/6 | 6/6 | 5/6 |
| 10 | *Burkholderia contaminans* AU24637 | 5/6 | 0/6 | 6/6 | 1/6 | 6/6 | 6/6 | 6/6 | 6/6 |
| 11 | *Burkholderia diffusa* AU1075 | 6/6 | 1/6 | 4/6 | 3/6 | 5/6 | 5/6 | 6/6 | 6/6 |
| 12 | *Burkholderia arboris* ES0263a | 6/6 | 0/6 | 3/6 | 0/6 | 5/6 | 0/6 | 6/6 | 6/6 |
| 13 | *Burkholderia arboris* AU22095 | 3/6 | 1/6 | 3/6 | 1/6 | 4/6 | 5/6 | 6/6 | 5/6 |
| 14 | *Burkholderia lata* HI4002 | 2/6 | 1/6 | 5/6 | 2/6 | 5/6 | 5/6 | 4/6 | 6/6 |
| 15 | *Burkholderia cenocepacia* AU1054 | 4/6 | 2/6 | 3/6 | 3/6 | 3/6 | 4/6 | 6/6 | 6/6 |
| 16 | *Burkholderia cenocepacia* AU0222 | 3/6 | 3/6 | 3/6 | 3/6 | 5/6 | 3/6 | 4/6 | 6/6 |
| 17 | *Burkholderia cenocepacia* AU19236 | 2/6 | 0/6 | 2/6 | 2/6 | 4/6 | 0/6 | 5/6 | 0/6 |
| 18 | *Burkholderia cenocepacia* HI2976 | 3/6 | 0/6 | 3/6 | 2/6 | 4/6 | 4/6 | 4/6 | 2/6 |
| 19 | *Burkholderia cenocepacia* HI2485 | 2/6 | 0/6 | 2/6 | 2/6 | 5/6 | 3/6 | 4/6 | 1/6 |
| 20 | *Burkholderia cenocepacia* J2315 | 3/6 | 1/6 | 3/6 | 4/6 | 4/6 | 2/6 | 4/6 | 6/6 |
| **Total** | | **70/120* (58.3%)** | **25/120 (20.8%)** | **62/120 (51.7%)** | **52/120 (43.3%)** | **75/120 (62.5%)** | **72/120 (60.0%)** | **84/120 (70.0%)** | **97/120 (80.8%)** |

[a]Number of positive/number of tests

*There is a statistically significant difference between the qPCR and LAMP methods ($p < 0.05$).

Table S4. Comparison of positive results for *B. cepacia* complex using LAMP and qPCR in CHX.

| | | Inoculum (CFU/ml) | | | | | | | |
|---|---|---|---|---|---|---|---|---|---|
| | | 10 | | $10^2$ | | $10^3$ | | $10^4$ | |
| | | LAMP | qPCR | LAMP | qPCR | LAMP | qPCR | LAMP | qPCR |
| 1 | *Burkholderia cepacia* PC783 | 0/6[a] | 0/6 | 1/6 | 0/6 | 5/6 | 0/6 | 5/6 | 5/6 |
| 2 | *Burkholderia cepacia* AU24442 | 2/6 | 0/6 | 0/6 | 0/6 | 2/6 | 0/6 | 4/6 | 3/6 |
| 3 | *Burkholderia stabilis* AU23340 | 1/6 | 0/6 | 1/6 | 2/6 | 0/6 | 2/6 | 1/6 | 5/6 |
| 4 | *Burkholderia pyrrocinia* AU11057 | 0/6 | 0/6 | 0/6 | 2/6 | 0/6 | 3/6 | 3/6 | 4/6 |
| 5 | *Burkholderia ambifaria* HI2468 | 1/6 | 0/6 | 0/6 | 0/6 | 0/6 | 3/6 | 0/6 | 5/6 |
| 6 | *Burkholderia anthina* HI2738 | 1/6 | 0/6 | 2/6 | 0/6 | 0/6 | 3/6 | 1/6 | 3/6 |
| 7 | *Burkholderia metallica* AU0553 | 0/6 | 0/6 | 2/6 | 0/6 | 3/6 | 3/6 | 4/6 | 5/6 |
| 8 | *Burkholderia metallica* AU16697 | 5/6 | 0/6 | 5/6 | 0/6 | 4/6 | 0/6 | 4/6 | 5/6 |
| 9 | *Burkholderia contaminans* HI3429 | 0/6 | 0/6 | 1/6 | 0/6 | 4/6 | 0/6 | 4/6 | 6/6 |
| 10 | *Burkholderia contaminans* AU24637 | 1/6 | 0/6 | 2/6 | 0/6 | 4/6 | 1/6 | 5/6 | 6/6 |
| 11 | *Burkholderia diffusa* AU1075 | 2/6 | 1/6 | 2/6 | 0/6 | 3/6 | 0/6 | 2/6 | 1/6 |
| 12 | *Burkholderia arboris* ES0263a | 3/6 | 0/6 | 1/6 | 0/6 | 2/6 | 0/6 | 2/6 | 2/6 |
| 13 | *Burkholderia arboris* AU22095 | 1/6 | 0/6 | 1/6 | 0/6 | 4/6 | 1/6 | 0/6 | 6/6 |
| 14 | *Burkholderia lata* HI4002 | 2/6 | 0/6 | 2/6 | 0/6 | 2/6 | 0/6 | 1/6 | 5/6 |
| 15 | *Burkholderia cenocepacia* AU1054 | 5/6 | 0/6 | 1/6 | 0/6 | 4/6 | 0/6 | 2/6 | 4/6 |
| 16 | *Burkholderia cenocepacia* AU0222 | 2/6 | 0/6 | 4/6 | 0/6 | 2/6 | 0/6 | 4/6 | 6/6 |
| 17 | *Burkholderia cenocepacia* AU19236 | 0/6 | 0/6 | 0/6 | 0/6 | 0/6 | 0/6 | 2/6 | 5/6 |
| 18 | *Burkholderia cenocepacia* HI2976 | 0/6 | 0/6 | 0/6 | 0/6 | 0/6 | 1/6 | 0/6 | 6/6 |
| 19 | *Burkholderia cenocepacia* HI2485 | 1/6 | 0/6 | 0/6 | 0/6 | 0/6 | 0/6 | 1/6 | 6/6 |
| 20 | *Burkholderia cenocepacia* J2315 | 2/6 | 0/6 | 1/6 | 0/6 | 0/6 | 2/6 | 4/6 | 2/6 |
| **Total** | | **29/120* (24.2%)** | **1/120 (0.83%)** | **26/120* (21.7%)** | **4/120 (3.3%)** | **39/120* (32.5%)** | **19/120 (15.8%)** | **49/120* (40.8%)** | **90/120 (75.0%)** |

[a]Number of positive/number of tests

*There is a statistically significant difference between the qPCR and LAMP methods ($p < 0.05$).

Table S5. Comparison of positive results for *B. cepacia* complex using LAMP and qPCR in BZK.

| | | Inoculum (CFU/ml) | | | | | | | |
|---|---|---|---|---|---|---|---|---|---|
| | | 10 | | $10^2$ | | $10^3$ | | $10^4$ | |
| | | LAMP | qPCR | LAMP | qPCR | LAMP | qPCR | LAMP | qPCR |
| 1 | *Burkholderia cepacia* PC783 | 0/6[a] | 0/6 | 0/6 | 0/6 | 2/6 | 4/6 | 4/6 | 0/6 |
| 2 | *Burkholderia cepacia* AU24442 | 1/6 | 0/6 | 4/6 | 4/6 | 4/6 | 6/6 | 5/6 | 6/6 |
| 3 | *Burkholderia stabilis* AU23340 | 3/6 | 0/6 | 3/6 | 3/6 | 2/6 | 4/6 | 4/6 | 3/6 |
| 4 | *Burkholderia pyrrocinia* AU11057 | 2/6 | 0/6 | 3/6 | 0/6 | 2/6 | 2/6 | 5/6 | 1/6 |
| 5 | *Burkholderia ambifaria* HI2468 | 3/6 | 0/6 | 1/6 | 0/6 | 3/6 | 4/6 | 2/6 | 4/6 |
| 6 | *Burkholderia anthina* HI2738 | 2/6 | 0/6 | 3/6 | 3/6 | 4/6 | 6/6 | 4/6 | 6/6 |
| 7 | *Burkholderia metallica* AU0553 | 2/6 | 0/6 | 2/6 | 4/6 | 6/6 | 6/6 | 4/6 | 6/6 |
| 8 | *Burkholderia metallica* AU16697 | 5/6 | 0/6 | 5/6 | 2/6 | 6/6 | 6/6 | 5/6 | 6/6 |
| 9 | *Burkholderia contaminans* HI3429 | 0/6 | 0/6 | 1/6 | 4/6 | 4/6 | 6/6 | 6/6 | 6/6 |
| 10 | *Burkholderia contaminans* AU24637 | 0/6 | 0/6 | 1/6 | 0/6 | 4/6 | 4/6 | 6/6 | 6/6 |
| 11 | *Burkholderia diffusa* AU1075 | 1/6 | 0/6 | 3/6 | 0/6 | 2/6 | 5/6 | 4/6 | 6/6 |
| 12 | *Burkholderia arboris* ES0263a | 2/6 | 0/6 | 1/6 | 1/6 | 2/6 | 4/6 | 4/6 | 6/6 |
| 13 | *Burkholderia arboris* AU22095 | 2/6 | 0/6 | 1/6 | 0/6 | 2/6 | 6/6 | 3/6 | 6/6 |
| 14 | *Burkholderia lata* HI4002 | 2/6 | 0/6 | 1/6 | 0/6 | 1/6 | 2/6 | 1/6 | 3/6 |
| 15 | *Burkholderia cenocepacia* AU1054 | 2/6 | 0/6 | 2/6 | 0/6 | 1/6 | 3/6 | 0/6 | 4/6 |
| 16 | *Burkholderia cenocepacia* AU0222 | 4/6 | 0/6 | 1/6 | 0/6 | 2/6 | 2/6 | 5/6 | 5/6 |
| 17 | *Burkholderia cenocepacia* AU19236 | 2/6 | 0/6 | 2/6 | 0/6 | 2/6 | 5/6 | 2/6 | 4/6 |
| 18 | *Burkholderia cenocepacia* HI2976 | 1/6 | 0/6 | 3/6 | 0/6 | 2/6 | 6/6 | 4/6 | 4/6 |
| 19 | *Burkholderia cenocepacia* HI2485 | 5/6 | 0/6 | 4/6 | 3/6 | 2/6 | 4/6 | 1/6 | 6/6 |
| 20 | *Burkholderia cenocepacia* J2315 | 3/6 | 0/6 | 4/6 | 2/6 | 4/6 | 6/6 | 0/6 | 6/6 |
| **Total** | | **42/120* (35.0%)** | **0/120 (0.0%)** | **45/120* (37.5%)** | **26/120 (21.7%)** | **57/120* (47.5%)** | **91/120 (75.8%)** | **69/120* (57.5%)** | **94/120 (78.3%)** |

[a]Number of positive/number of tests

*There is a statistically significant difference between the qPCR and LAMP methods ($p < 0.05$).

Figure S1. Venn diagram for the clusters of BCC group and non-BCC group. Among 174,715 orthologous gene clusters (with ≥ 75% sequence identity) observed across all 266 complete *Burkholderia* genomes, 206 BCC-specific gene clusters were identified (a) and work flow of primer design process (b).

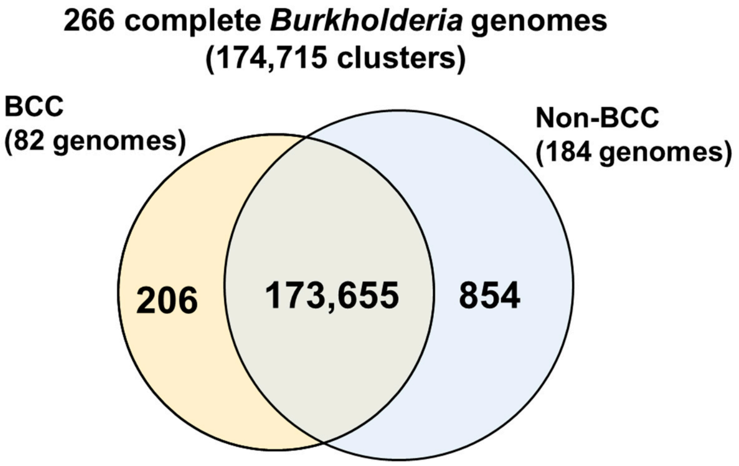


(a)

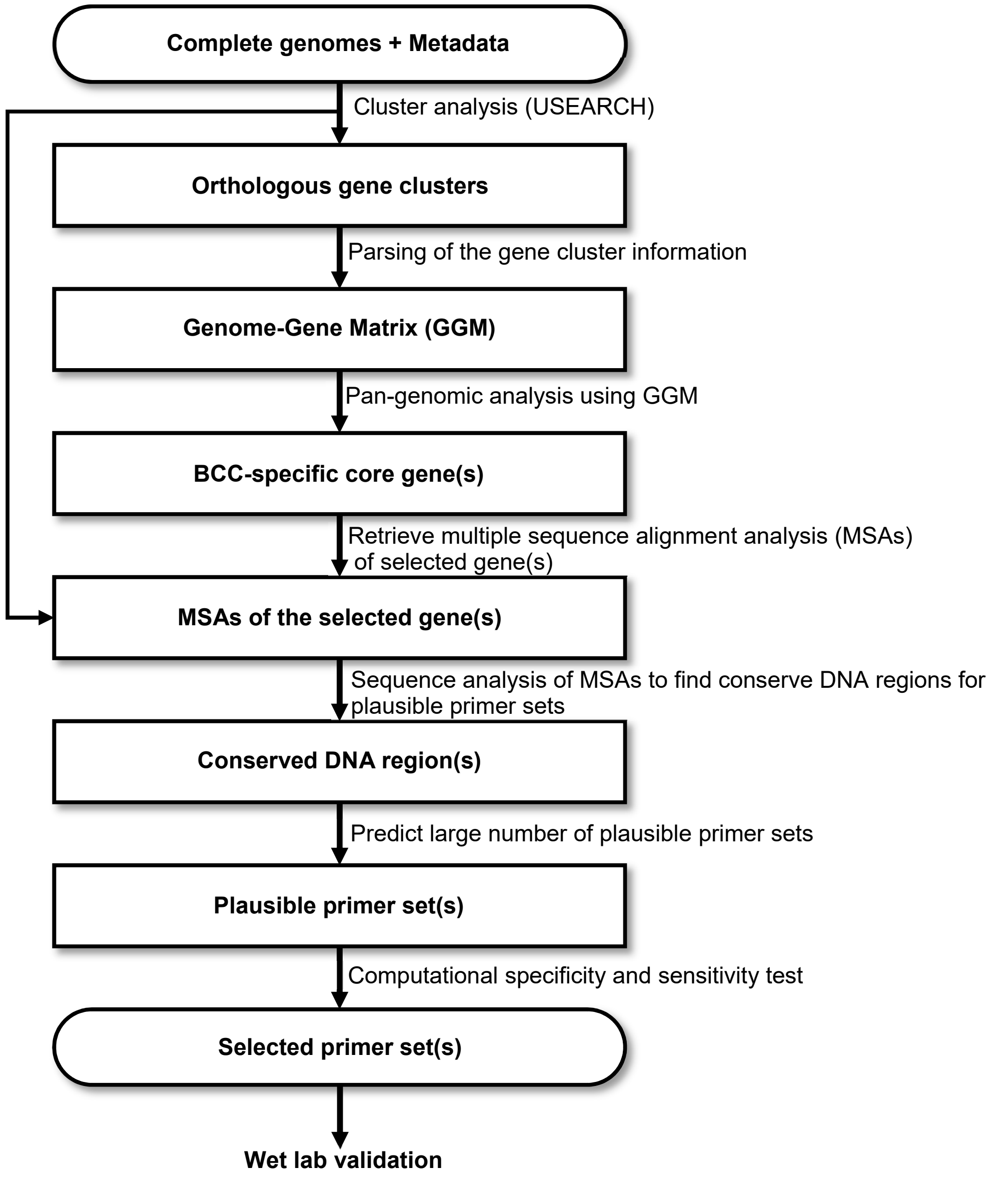
Complete genomes + Metadata
Cluster analysis (USEARCH)
Orthologous gene clusters
Parsing of the gene cluster information
Genome-Gene Matrix (GGM)
Pan-genomic analysis using GGM
BCC-specific core gene(s)
Retrieve multiple sequence alignment analysis (MSAs) of selected gene(s)
MSAs of the selected gene(s)
Sequence analysis of MSAs to find conserve DNA regions for plausible primer sets
Conserved DNA region(s)
Predict large number of plausible primer sets
Plausible primer set(s)
Computational specificity and sensitivity test
Selected primer set(s)
Wet lab validation

(b)

Figure S2. Different reaction volume conditions (5, 10, 15, and 20 µl) of LAMP assay using 10.3 µg/ml and 1.03 µg/ml of *B. cenocepacia* AU1054 at 65°C. Colorimetric LAMP for the detection of BCC using RibB 16 primer.

10.3 µg/ml

1.03 µg/ml

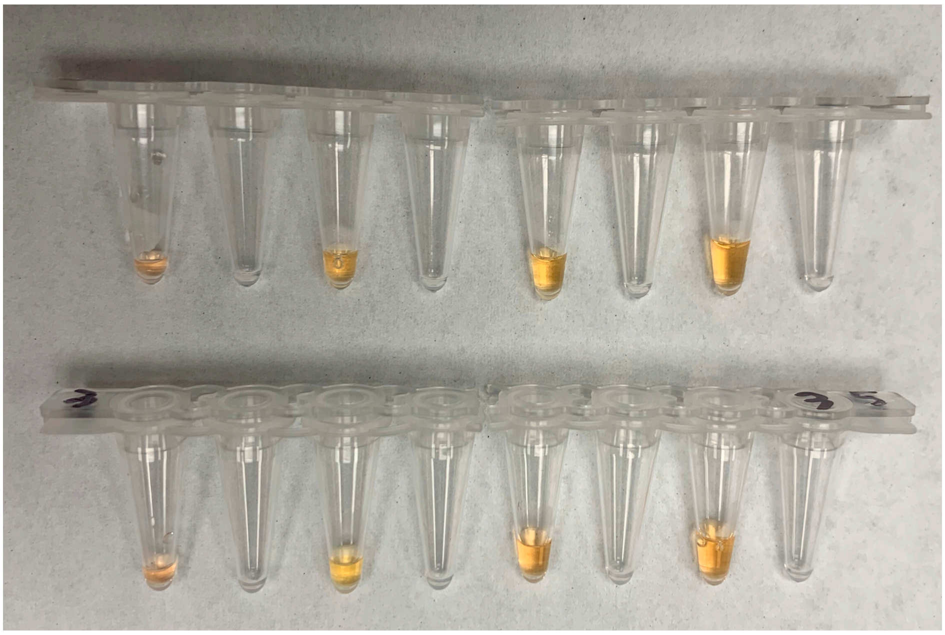

5 µl 10 µl 15 µl 20 µl
